# Oroxylin A suppresses the development and growth of colorectal cancer through reprogram of HIF1*α*-modulated fatty acid metabolism

**DOI:** 10.1038/cddis.2017.261

**Published:** 2017-06-08

**Authors:** Ting Ni, Zihao He, Yuanyuan Dai, Jingyue Yao, Qinglong Guo, Libin Wei

**Affiliations:** 1 State Key Laboratory of Natural Medicines, Jiangsu Key Laboratory of Carcinogenesis and Intervention, Department of Basic Medicine, School of Basic Medicine and Clinical Pharmacy, China Pharmaceutical University, 24 Tongjiaxiang, Nanjing, China

## Abstract

The occurrence and progress of colon cancer are closely associated with obesity. Therefore, the lipid metabolism, especially fatty acid metabolism, is a significant section of energy homeostasis in colon cancer cells, and it affects many important cellular processes. Oroxylin A is one of the main bioactive flavonoids of Scutellariae radix, which has a strong anticancer effect but low toxicity to normal tissue. In previous studies, we have proved that oroxylin A reprogrammes metabolism of cancer cells by inhibiting glycolysis. Here, we further investigated the metabolism-modulating effects of oroxylin A on the fatty acid metabolism in colon cancer cells under hypoxia. We found that HIF1*α* upregulated adipophilin, fatty acid synthase and sterol regulatory element-binding protein 1, and downregulated carnitine palmitoyltransferase 1 (CPT1), resulting in the promoted lipid uptake and transport, increased *de novo* fatty acid synthesis and suppressed fatty acid oxidation. Oroxylin A inactivated HIF1*α* and reprogrammed fatty acid metabolism of HCT116 cells, decreasing intracellular fatty acid level and enhancing fatty acid oxidation. Furthermore, the rapid decrease of fatty acid level caused by oroxylin A inhibited the nuclear translocation of *β*-cantenin and inactivated the Wnt pathway, arousing cell cycle arrest in G2/M phase. *In vivo* studies demonstrated that high-fat diet increased the incidence of colon cancer and accelerated tumor development. Importantly, besides the growth inhibitory effects on colon cancer xenograft, oroxylin A prevented carcinogenesis and delayed progress of primary colon cancer as well. Our studies enriched the metabolic regulatory mechanism of oroxylin A, and suggested that oroxylin A was a potent candidate for the treatment and prevention of colorectal cancer.

The surplus amount of lipids in obesity is one of the biggest public health problems facing the world today. It is predicted that by 2025 over 700 million people will be either overweight or obese worldwide. Obesity heightens the risk of several chronic and potentially life-threatening illnesses, including cancer development.^1, 2^ According to the report of World Cancer Research Fund (WRCF), obesity may account for 25–30% of major cancers, such as colon, breast, gallbladder, ovaries, pancreas, kidney and cancer of the esophagus.^3^ There is a clear, direct link between obesity and colorectal cancer. Latest studies show that obesity primes cancer risk, and obese individuals have a 20% greater risk of developing colorectal cancer compared with those of normal weight.^4^ The analyses also indicated that obese men are at 30% greater risk of developing the cancer compared with obese women. Numerous observations in mice, cell culture models and obese individuals have shown that lipid accumulation is associated with tumor development. However, we need more experimental verification to determine the exact role of this metabolic alteration in the context of cancer and to find the key regulator.

Cellular energy metabolism dysfunction is an important feature of almost all cancers regardless of cellular or tissue origin. In contrast to normal cells, which primarily rely on mitochondrial oxidative phosphorylation (OXPHOS) to generate energy, most malignant cells instead rely on aerobic glycolysis, a phenomenon termed ‘the Warburg effect’.^5, 6^ Limitations in tumor vascularization result in periods of intermittent hypoxia that force cells to rely on glycolysis, producing energy and providing a survival advantage for tumor cells.^7^ The elevated glucose catabolism produces an excess of pyruvate, most of which is converted to lactate, being benefited to the balance of the tumor microenvironment. However, some of the pyruvate is converted to acetyl-CoA, which, in turn, is used in *de novo* fatty acid synthesis.^8^ Highly proliferating cancer cells need to synthesize fatty acids *de novo* to continually provide lipids for membrane production and energy production through *β*-oxidation and lipid modification of proteins, which act as second messengers regulating signaling pathways, as well as participating in protein post-translational modification.^9, 10^ Just a small shift in the regulation of lipid metabolism can lead to a large change in energy homeostasis; it can result in excess fat accumulation and it may also affect many important cellular processes, including cell growth, proliferation and differentiation.^11^

Lipid metabolism is complicated, involving a large number of enzymes catalyzing metabolic reactions with regulation at different levels, such as lipid uptake, fatty acid transport, fatty acid synthesis and oxidation.^12^ The level and synthesis of fatty acids, phospholipids and cholesterol were significantly increased in tumor cells. And fatty acids’ metabolism was most widely studied. Many genes related with *de novo* fatty acid synthesis were highly expressed in cancer cells and were associated with a variety of malignant phenotypes.^13^ Acetyl-CoA carboxylase (ACC) and fatty acid synthase (FASN) were key enzymes involving *de novo* fatty acid synthesis, which were both the targeted gene of sterol regulatory element-binding proteins (SREBP). It was reported that PI3K/AKT and hypoxia-inducible factor 1*α* (HIF1*α*) could influence fatty acid metabolism by modulating SREBP.^8, 14^ Hypoxia has a major role in metabolic reprogramming of tumor cells. The hypoxic environment of a tumor leads to stabilization of the HIF1*α*, which stimulated glycolysis through induction of glucose transporters, hexokinase II (HKII) and lactate dehydrogenase A, and inhibited mitochondrial respiration by pyruvate dehydrogenase kinase 1 (PDK1).^15^ Glycolysis promoted by HIF1*α* provided abundant precursors for fatty acid synthesis. Moreover, HIF1*α* enhanced fatty acid uptake and accumulation of lipid droplets (LDs),^16^ and suppressed fatty acid oxidation (FAO) by downregulating long-chain acyl-CoA dehydrogenases.^17^

Oroxylin A (OA), an active component of a Chinese traditional medicinal plant Scutellaria baicalensis Georgi, showed strong anticancer effects by reprogramming glycolytic metabolism of cancer cells in previous studies.^18, 19^ Here, we further explored the effects of OA in modulating the lipid metabolism of colon cancer. Most previous reports involving the anti-colon cancer effects of OA were associated with inflammation,^20, 21^ and they ignored another important factor that was excess fat. In this study, we demonstrated fatty acid metabolism-facilitated colon cancer progression, and found that OA modulated fatty acid metabolism through HIF1*α*, inducing the inactivation of the Wnt signaling pathway and cell growth inhibition of colon cancer.

## Results

### Oroxylin A modulated lipid metabolism of colon cancer cells stimulated with PA under hypoxia

**Figure 1:**
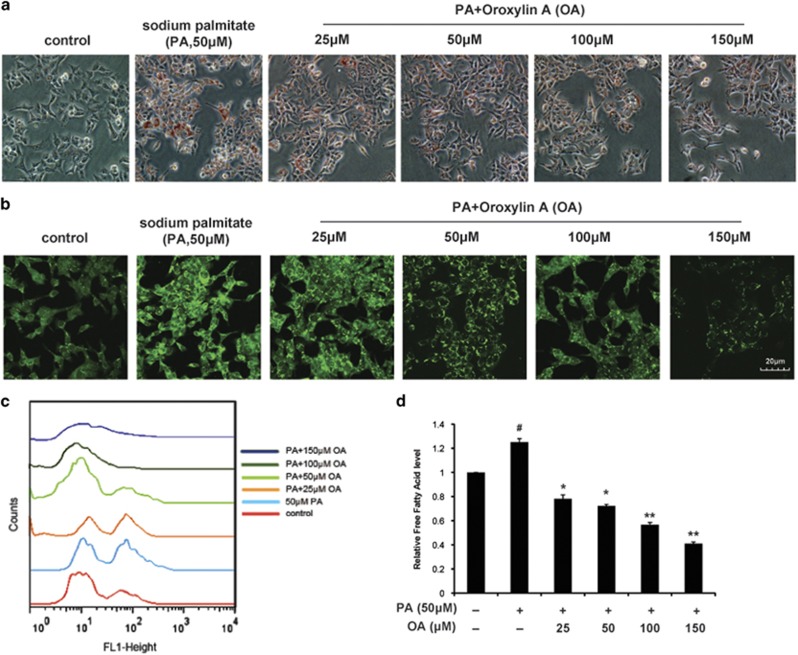
OA decreased the intracellular lipid level of colon cancer cells under hypoxia. HCT116 cells were cultured in the McCoy’s 5A culture medium containing 10% FBS and 50 *μ*M sodium palmitate (PA) under hypoxia. Cells were treated with OA (25, 50, 100 and 150 *μ*M) for 36 h and then collected. (**a**) Cells were stained with oil red and observed. (**b**) Cells were stained with nile red and observed under confocal microscopy (× 400). (**c**) Cells were stained with nile red. Then fluorescence intensity was quantified by flow cytometry at Ex/Em of 552/636 nm. (**d**) The intracellular free fatty acid was assayed with the Free Fatty Acid Quantification Kit. Bars, S.D.; ^#^*P*<0.05 *versus* untreated controls in hypoxia; *P<0.05 or **P<0.01 *versus* PA treated alone in hypoxia

HCT116 cells were cultured in the McCoy’s 5A culture medium containing 10% FBS and 50 *μ*M sodium palmitate (PA). By oil red staining and nile red staining, we found that hypoxia condition induced lipid droplet (LD) accumulation (Supplementary Figures S1A and S1B). Upon OA treatment, the LD accumulation was suppressed significantly (Figures 1a and b). The flow cytometry assay of HCT116 staining with nile red showed that the level of cellular neutral lipid was decreased (Figure 1c). And the total content of cellular free fatty acid was measured, and it was seen to decrease as well (Figure 1d).

### HIF1*α* is a key controller of oroxylin A-induced modulation of fatty acid metabolism

**Figure 2:**
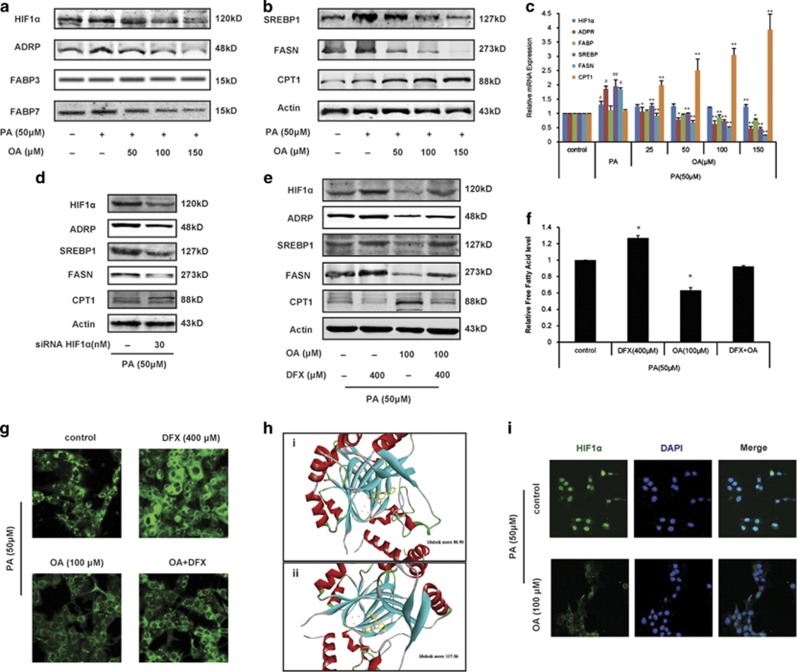
OA reprograms HIF1*α*-modulated fatty acid metabolism of colon cancer. (**a**–**c**) HCT116 cells were cultured in the McCoy’s 5A culture medium containing 10% FBS and 50 *μ*M PA, and treated with OA for 36 h under hypoxic conditions. Western blot (**a**, **b**) and quantitative RT-PCR assays (**c**) were performed for HIF1*α* and fatty acid metabolism-related proteins, including ADRP, FABP3, FABP7, SREBP1, FASN and CPT1. (**d**) Western blot was performed for fatty acid metabolism-related proteins in HIF1α-deficient HCT116 cells. (**e**–**g**) HCT116 cells were cotreated with OA and DFX (HIF1*α* inducer) for 36 h. Fatty acid metabolism-related proteins level (**e**), intracellular free fatty acid level (**f**) and neutral lipid (**g**) were assayed. (**h**) Binding mode of the OA (i) and YC-1 (ii) from virtual screening with HIF1*α*. (**i**) The colocalization of HIF1*α* with nucleus was performed by immunofluorescence using antibodies specific to HIF1*α* and DAPI (× 400). Bars, S.D.; ^#^*P*<0.05 or ^##^*P*<0.01 *versus* untreated controls in hypoxia; *P<0.05 or **P<0.01 *versus* PA treated alone in hypoxia

Under hypoxia, fatty acid-binding protein (FABP), adipophilin (ADRP, an essential LD structural component), FASN and its transcription factor sterol regulatory element-binding protein 1 (Srebp1) were all enhanced (Supplementary Figures S1C and S1D). However, the rate-limiting enzyme of *β* oxidation carnitine palmitoyltransferase was decreased. These results suggested that hypoxia could promote LD formation and the accumulation of fatty acid, but it instead inhibited FAO. After HCT116 cells were treated with OA, LD formation was impaired, showing that the protein expressions and mRNA expressions of ADRP, Srebp1 and FASN were significantly decreased, but those of CPT1 were increased (Figures 2a–c). The downregulation of FABP gene was observed upon high concentration of OA (150 *μ*M), and only the protein level of subtype FABP7 was decreased. Besides, OA downregulated HIF1*α* protein level, without influencing HIF1*α* mRNA level. OA reprogrammed fatty acid metabolism in a multi-link manner, including the uptake, transportation, synthesis and oxidation of fatty acid.

After HIF1*α* was knocked down, ADRP, Srebp1 and FASN were downregulated, while the level of CPT1 was increased(Figure 2d), suggesting that HIF1*α* was an important modulator of LD formation and fatty acid metabolism under hypoxia. When HTC116 cells transfected with HIF1*α* siRNA were treated with OA, the influences to intracellular fatty acid level and HIF1*α*-targeted fatty acid-regulating enzymes were strengthened (Supplementary Figures S2A and S2B). Then, we cotreated HCT116 cells with OA and desferrioxamine (DFX, HIF1*α* inducer),^22^ and found that the impaired effect for LD formation and the reprogramming of fatty acid metabolism caused by OA was weakened by DFX (Figures 2e and f). In further studies, we found that OA could bind with HIF1*α*, being a potential HIF1*α* inhibitor (Figure 2g). Compared with HIF1*α* inhibitor YC-1, OA interacted with the pocket structure of HIF1*α* protein, which was formed by Phe100, Tyr102, Gln147, Tyr145, Leu188, Thr196, Asp201, Asn205, Phe207 and other residues. The combination of hydrophobic interaction was the main driving force. Hydrogen atom of hydroxyl acted as a hydrogen bond acceptor to form hydrogen bonds with the His279 residues. Moreover, the nuclear translocation of HIF1*α* was suppressed by OA (Figure 2h).

The above results demonstrated that OA impaired LD formation and reprogrammed fatty acid metabolism of colon cancer cell by inactivating HIF1*α*, which was the key regulator for fatty acid metabolism under hypoxia.

### Oroxylin A induces G2/M cell cycle arrest and suppresses the growth of colon cancer cells stimulated with PA under hypoxia

**Figure 3:**
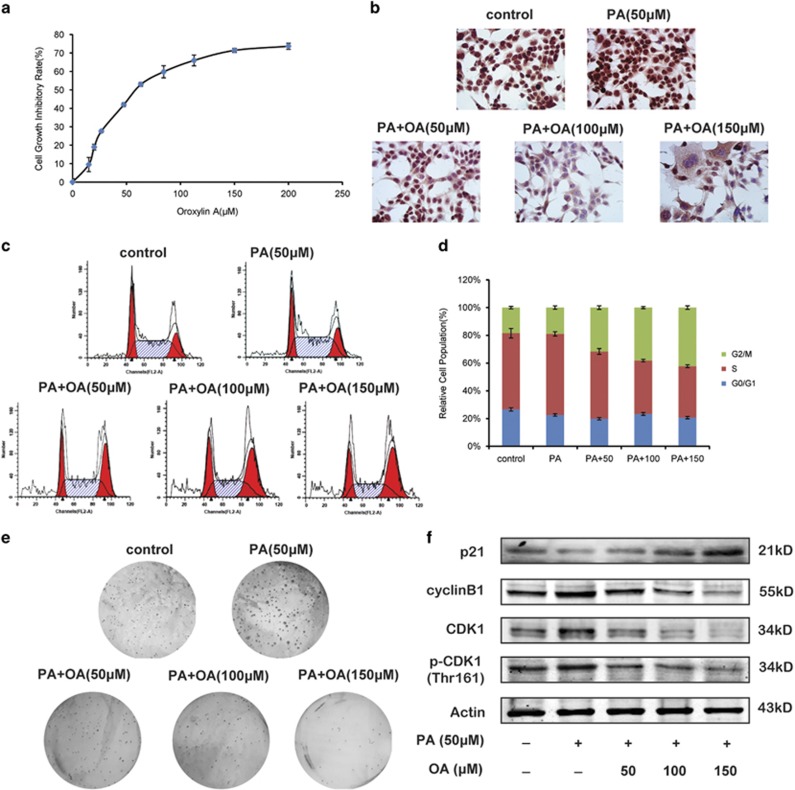
OA induces cell cycle arrest and suppresses the growth of colon cancer cells under hypoxia. HCT116 cells were cultured in the McCoy’s 5A culture medium containing 10% FBS and 50 *μ*M PA, and treated with OA for 36 h under hypoxia. (**a**) Cell growth inhibitory rates were measured by MTT assay. (**b**) Cells were fixed, permeabilized and stained with Ki67 assay kit. (**c**) Cell cycle distribution was monitored by flow cytometry. (**d**) The cell cycle distributions were quantified. (**e**) The colony formation ability was investigated after culturing at 37 °C, 5% CO_2_ for 28 days. The pictures of colonies in a six-well dish were taken with phase contrast microscopy. (**f**) Western blot assays were performed for cycle-related protein, including p21, cyclin B, CDK1 and p-CDK1 (Thr161)

In previous studies, we found that OA induced apoptosis of HCT116 cells.^23^ Here, HCT116 cells were prestimulated with 50 *μ*M PA. The results of MTT assay and Ki67 staining showed that OA inhibited the growth of HCT116 cells with the IC50 value of 38.7 *μ*M under hypoxia (Figures 3a and b). Moreover, OA induced cell cycle arrest at G2/M phase in a concentration-dependent manner (Figures 3c and d). We found that in OA-treated HCT116 cells, the protein expression of p21 was increased, and that of cyclin B1 was decreased (Figure 3f). Besides, the activation of CDK1 was inhibited by OA (Figure 3f). The colony formation of HCT116 cells was suppressed by OA as well (Figure 3e).

### The level of intracellular fatty acid influences canonical Wnt pathway, and is important for the growth inhibitory effect of oroxylin A

**Figure 4:**
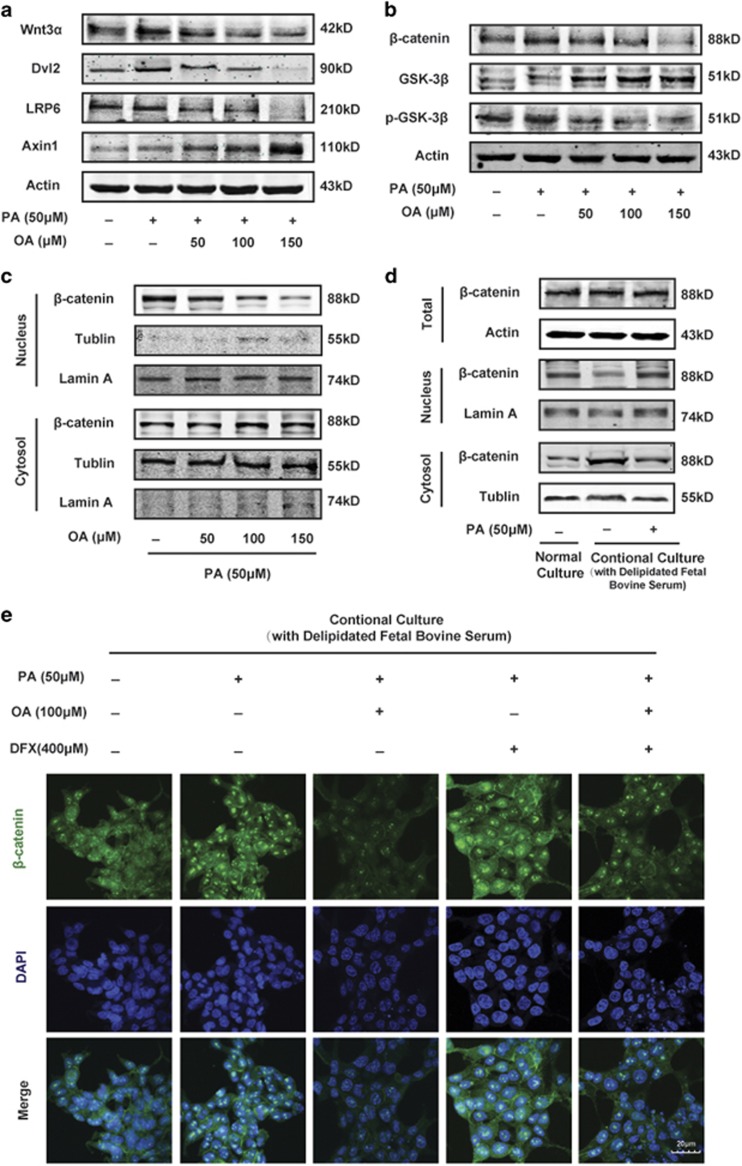
The effects of OA on the canonical Wnt pathway are associated with intracellular free fatty acid level. (**a**–**c**) HCT116 cells were cultured in the McCoy’s 5A culture medium containing 10% FBS and 50 *μ*M PA, and treated with OA for 36 h under hypoxia. Western blot assays were performed for Wnt3a, Dvl2, LRP6, Axin (A), total *β*-catenin, GSK-3*β*, p-GSK-3*β* (S9) (**b**) and *β*-catenin level in the cytoplasm and in the nucleus. (**d**, **e**) HCT116 cells were cultured in the McCoy’s 5A culture medium with 10% delipidated FBS, adding with or without PA. (**d**) Western blot assays were performed for total *β*-catenin and *β*-catenin in the cytoplasm as well as in the nucleus. (**e**) Cells were cotreated with OA and DFX. The nuclear localization of *β*-catenin was performed by immunofluorescence using antibodies specific to *β*-catenin and DAPI (× 400)

The canonical Wnt signaling pathway has emerged as a critical regulator pathway of cell proliferation and differentiation. Therefore, we investigated the effect of OA on Wnt signaling pathway in HCT116 cells stimulated with PA. Western blot analysis showed OA that decreased the protein levels of Wnt3a and its receptor LRP6 as well as Disheveled (Dvl2) (Figure 4a). Upon OA treatment, the downregulation of *β*-catenin was accompanied by increased level of Glycogen synthase kinase 3*β* (GSK-3*β*) and AXIN (Figure 4b). The inactive form of GSK-3*β*, p-GSK-3*β* (S9), was decreased, indicating that destruction complex was activated to phosphorylate *β*-catenin for proteasomal degradation (Figure 4b). Moreover, OA changed the intracellular distribution of *β*-catenin, which was remarkably decreased in the nucleus (Figure 4c).

We had found that OA suppressed fatty acid metabolism and inhibited cell growth of HCT116 cells. To explore the relationship between the two effects of OA, we took up the conditional culture by using delipidated FBS. When the lipid in the culture medium was deprived, the *β*-catenin level in the nucleus was decreased, and the adding of exogenous fatty acid could reverse the transportation of *β*-catenin from the nucleus to the cytoplasm (Figure 4d). Moreover, we knocked down FASN to decrease the fatty acid level (Supplementary Figure S3A), resulting in the inhibition of cell growth and the decrease of *β*-catenin level in the nucleus (Supplementary Figure S3B and S3C). These results suggested that the intracellular fatty acid level would influence the distribution of *β*-catenin. Then, the result of fluorescent colocation assay also showed that PA promoted the nuclear transportation of *β*-catenin, whereas OA inhibited it (Figure 4e). Besides, DFX reversed the effect of OA as well. When HCT116 cells overexpressed FASN, the cell growth inhibition and the suppression of *β*-catenin nuclear translocation induced by OA were both weakened (Supplementary Figures S3D and S3E). In all, the level of cellular fatty acid is important for the growth inhibitory effect of OA by inhibiting the nuclear translocation of *β*-catenin.

### The antiproliferation activity of oroxylin A in colon cancer xenograft is related with the influence of fatty acid metabolism

**Figure 5:**
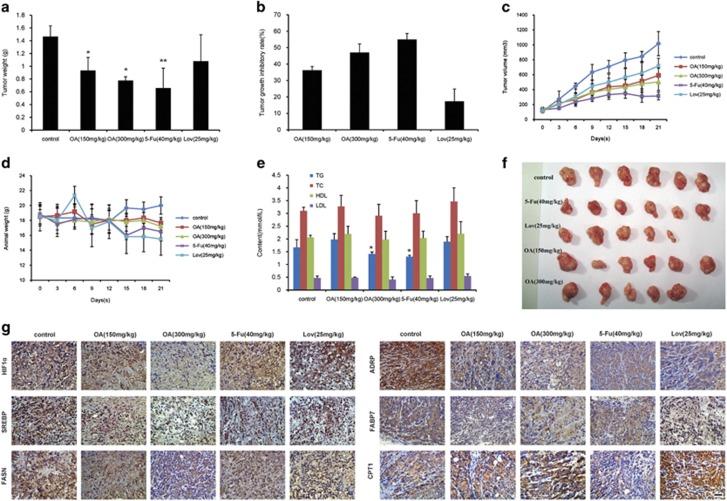
OA inhibited the growth of transplanted human colon tumor. Nude mice inoculated with HCT116 cells were treated with saline control, oroxylin A, 5-Fu and lovastatin. The tumor weight (**a**), tumor volume (**c**) and animal weight (**d**) were measured. (**b**) The tumor inhibitory rates were calculated. (**e**) The triglyceride (TG), total cholesterol (TC), low-density lipoprotein (LDL) and high-density lipoprotein (HDL) in the blood were assayed. (**f**) Photographs for tumor. (**g**) Expressions of HIF1*α* and fatty acid metabolism-related proteins in the central parts of colon tumor tissue were assessed by immunohistochemistry, scale bar=20 *μ*m. Bars, S.D.; *P<0.05 *versus* control group

In further studies, we investigated the effect of OA in a xenograft model of nude mice inoculated with HCT116 cells. OA concentrations of 150 and 300 mg/kg inhibited the growth of tumor significantly, with inhibitory rates of 36.3±2.33% and 47.0±5.35%, respectively (Figures 5a–c and f). An OA concentration of 300 mg/kg (p.o.) was as effective as 40 mg/kg 5-Fu intravenous (i.v.). We also tested the anticancer effect of lovastatin, which is an important statin in clinical studies. Disappointing for us, the tumor inhibitory rate of 25 mg/kg lovastatin was lower than 20%. During the experiment, OA had little influence on the animal weight. Instead, 5-Fu and lovastatin significantly decreased animal weight during the latter period of the experiment. By testing the blood fat of mouse, we found that OA could decrease the level of triglyceride (TG) (Figure 5e). The IHC assay for the center part of tumor tissue was performed, which had a lack of oxygen and blood vessels. The result showed that 300 mg/kg OA decreased the levels of HIF1*α*, Srebp1, FASN, ADRP and FABP7, and increased the CPT1 level (Figure 5g). 5-Fu concentration of 40 mg/kg did not show effective regulation on the fatty acid of colon cancer tissue. Besides, 25 mg/kg lovastatin significantly decreased the ADRP level and increased the CPT1 level; however, it had little influence on HIF1*α* and other factors.

These results demonstrated that growth inhibition of colorectal cancer xenograft induced by OA was related with the modulation of fatty acid metabolism.

### Oroxylin A suppressed the development of primary colorectal cancer by reprogramming fatty acid metabolism *in vivo*

Finally, we established AMO/DSS-induced primary colon cancer in C57BL/6 mice and investigated the role of fatty acid metabolism in the development of colon cancer.

**Figure 6:**
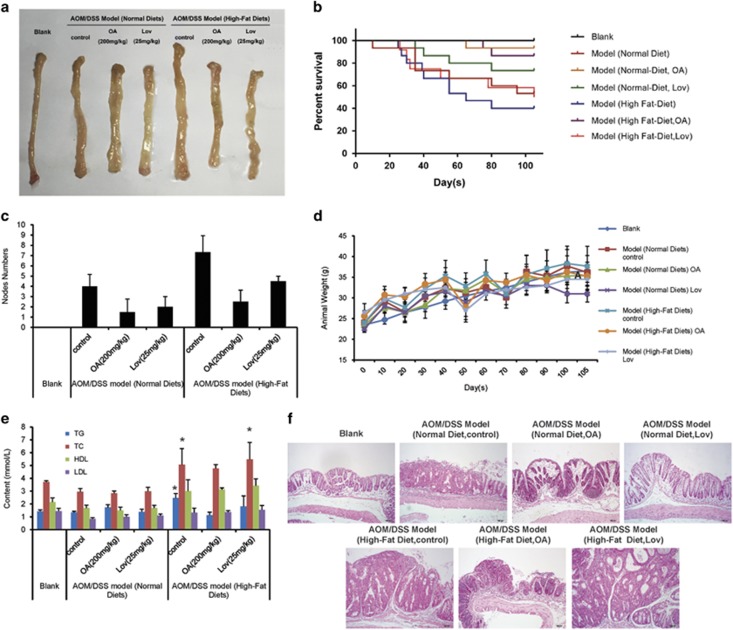
High-fat diet promotes the incidence and development of colon cancer, and OA reduced the cancer progression. C57BL/6 mice were subjected to an AOM-based CAC induction protocol using three cycles of 2.5% DSS in drinking water. In the beginning of the model, the mice were divided into two big groups, and administrated with normal diet and high-fat diet, respectively. Mice were treated with OA or lovastatin in the beginning of the experiment. (**a**) Representative images of colons removed from mice on day 105. (**b**) Kaplan–Meier survival curves show the effect of oroxylin A on the survival of mice. (**c**) Tumor numbers were counted on day 105. (**d**) Body weights of mice were measured. (**e**) The triglyceride (TG), total cholesterol (TC), low-density lipoprotein (LDL) and high-density lipoprotein (HDL) in the blood were assayed. (**f**) H&E stains of serial sections of colons, × 100 magnification; scale bar=100 *μ*m. Bars, S.D.; *P<0.05 *versus* blank group

In the beginning of the experiment, the mice were divided into two main groups and fed with normal diet and high-fat diet, respectively. In each main group, the mice were administrated with 200 mg/kg OA or 25 mg/kg lovastatin. As shown in Figure 6a, we observed edema and nodes in the rectum wall of mice in model groups. Besides, we found blood in the stool of AOM/DSS model, especially in high-fat diet AOM/DSS model during the experiment. Kaplan–Meier survival curves showed that OA treatment significantly increased the survival of AOM/DSS-treated mice during the experiment (Figure 6b). Lovastatin showed protective effect to some degree, but it was much weaker than OA. Assessment of tumor number at the end of the animal experiment showed that high-fat diet promoted tumorigenesis and OA reduced tumor number in AOM/DSS model administrated with normal diet and high-fat diet (Figure 6c). The body weights of mice were monitored throughout the study, and the results showed that animals got fatter upon high-fat diet, but they lost weight after each exposure to lovastatin (Figure 5d). OA regained the body weight of AOM/DSS mice administrated with normal diet and kept the weight steady. The blood fat testing showed that OA controlled the TG level (Figure 5e). Histological examination of colonic sections was performed to assess intestinal cancerous status. As shown in Figure 5f, the results of hematoxylin and eosin (H&E) staining showed that samples of AOM/DSS model administrated with normal diet had a large adenocarcinoma inside the lumen, whose cells exhibited cylindrical shape, large nuclei, increasing nuclear/cytoplasmic (N/C) ratio and cellular cleavage, and the glands had abnormal sizes and shapes. The samples of AOM/DSS model administrated with high-fat diet showed that the cancer was poorly differentiated and invaded into the mucous membrane, accompanied by degeneration and necrosis of crypt cells and an amount of infiltrative inflammation. OA relieved these symptoms significantly, showing only moderate edema in lamina propria interstitial or mild inflammatory cell infiltration. Lovastatin had similar effects, but it showed higher tumor incidence than OA did.

**Figure 7:**
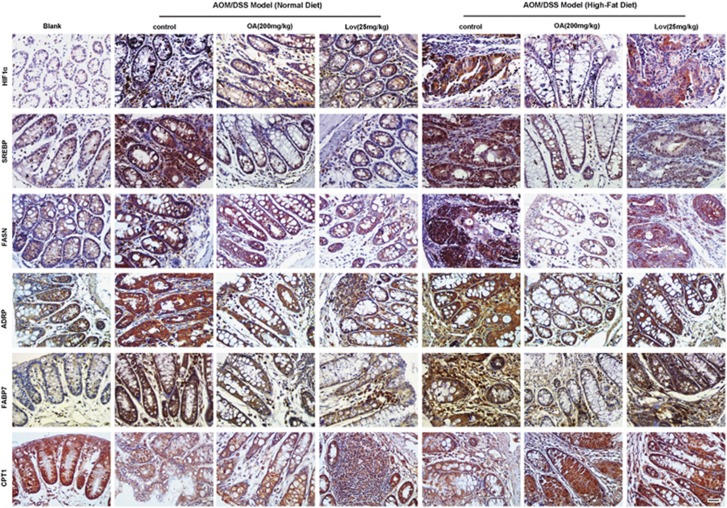
High-fat diet influences the fatty acid metabolism of primary colon cancer and OA reprograms this metabolic process. Expression of HIF1*α* and fatty acid metabolism-related proteins in tumor tissues of mice was examined by immunohistochemistry. Scale bar=20 *μ*m

By IHC assay, we found that the levels of HIF1*α*, Srebp1, FASN, ADRP and FABP7 were significantly enhanced, and the CPT1 level was decreased in the mice colon tissue of AOM/DSS model compared with the normal colon tissue(Figure 7). In the group of AOM/DSS model administrated with high-fat diet, the expressions of HIF1*α*, Srebp1, FASN and ADRP were much higher than those administrated with normal diet. OA could decrease HIF1*α*, Srebp1, FASN, ADRP and FABP7 levels, and increase CPT1 level. However, the effects of lovastatin in the model mice administrated with high-fat diet were not as strong as OA, especially in the suppression of HIF1*α*, ADRP and FABP7. Moreover, the influences of OA on the levels of HIF1*α*, FASN and CPT1 in the high-fat diet group were more obvious than those in the normal diet group, suggesting that high activity of lipid metabolism made colon cancer cells more sensitive to OA.

Taken together, these results indicated that high-fat diet increased incidence of colon cancer and accelerated tumor development, and OA inhibited carcinogenesis and tumor development in AOM/DSS mouse model.

## Discussion

**Figure 8:**
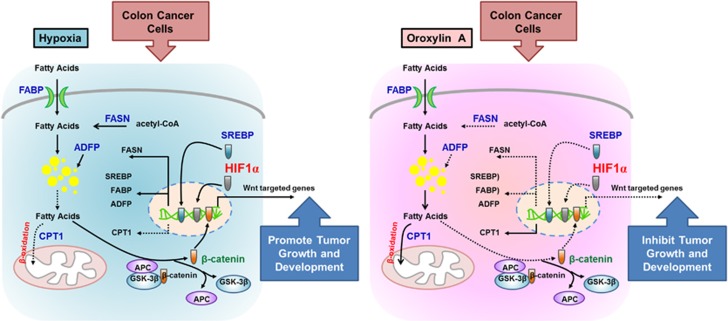
Schematic diagram of OA-induced growth inhibition of colon cancer via reprogramming fatty acid metabolism under hypoxia. Under hypoxia, activated HIF1*α* promoted the transcription of fatty acid metabolism-related protein FABP, ADRP and FASN, and decreased the CPT1 expression, resulting in the enhanced fatty acid level and lipid accumulation. The high level of free fatty acid activated the canonical Wnt pathway and promoted the nuclear translocation of *β*-catenin, facilitating colon cancer growth. OA inhibited HIF1*α*-mediated reprogram of fatty acid metabolism and inactivated the Wnt pathway, leading to the suppression of cell growth and development of colon cancer

Lipid metabolism is regulated by a complex signaling network. Increased *de novo* fatty acid synthesis plays significant roles in cancer pathogenesis. In this study, we found that increased fatty acid level promoted the growth of colon cancer and accelerated cancer development through activating Wnt/*β*-catenin pathway. Natural flavonoids OA could reduce intracellular fatty acid level by decreasing lipid uptake, suppressing LD formation and *de novo* fatty acid synthesis and enhancing FAO. The rapid decrease of fatty acid level inhibited the nuclear translocation of *β*-cantenin and inactivated Wnt pathway, resulting in G2/M cell cycle arrest and cell growth inhibition under hypoxia (Figure 8).

The role of lipid metabolism in the development and progression of cancer has been investigated in many types of cancer, such as colon cancer, prostate cancer and liver cancer. And the crucial importance of lipids, including fatty acids, phospholipids and cholesterol, in the expression of the malignant phenotype is reported.^24, 25, 26^ In general, phospholipids provided raw materials for the biological membrane formation of rapidly proliferating cancer cells, fatty acids produced reserve energy in the harsh environment, and cholesterol was involved in the movement and metastasis of malignant cell. However, the mechanism of lipids in facilitating the growth and development of cancer is not fully understood. Is there something special in the relationship between cellular lipids and tumor formation? The synthesis and oxidation of fatty acid were the focus of lipid metabolism studies.

The FASN complex facilitates lipogenesis by synthesizing palmitate from its base components. FASN expression in normal adult tissues is generally very low or undetectable, and it is significantly upregulated and correlates with poor prognosis in many types of cancer.^8^ Recent data demonstrated that FASN was a metabolic oncogene and its expression was important for tumor growth and survival.^27^ It was reported that the resistance to chemo- or radiotherapy of breast cancer and pancreatic cancer was closely related to FASN expression level.^28, 29^ Therefore, FASN is a promising anticancer target that may result in chemosensitization or enhanced efficacy when FASN function is disrupted as part of combination therapy. To date, several FASN inhibitors have shown antitumor activity including cerulenin, C75, orlistat, C93, GSK 837149A and natural plant-derived polyphenols.^27^ Cerulenin, a natural compound isolated from Cephalosporium caerulens, and its derivative C75 reacted with FASN to inhibit its activity, inducing cancer cell apoptosis by p53 accumulation, induction of ER stress and so on.^30, 31^ When FASN was knocked down, the cell growth of colon cancer cells and their nucleus translocation of *β*-cantenin were inhibited. Natural compound OA could decrease FASN expression and induce cell cycle arrest under hypoxia.

Generally, cancer cells adopt a *de novo* lipid synthesis strategy rather than use of stored lipids, resulting in the accumulation of LDs. Therefore, reduced lipid catabolism in cancer cells is expected to be of benefit to sustain rapid cell proliferation. However, recent results did not seem to be in agreement with this expectation, and contradictory consequences existed in the fatty acid catabolism for cancer progression. Hypoxia is a hallmark for cancer cells, and HIF1*α* is largely responsible for alterations in metabolism that support the survival of hypoxic tumor cells. HIF1*α* not only regulated FASN expression and facilitated fatty acid synthesis as well as lipid storage but also had significant regulation on FAO. It was reported that hypoxia diminished FAO in cultured cardiac myocytes in a HIF1-dependent manner,^32^ as well as in some cancer cells.^17, 33^ Under hypoxia, glycolysis was the predominant manner to produce energy in most cancer. When glycolysis was suppressed, FAO became the emergency power source and generated acetyl-CoA. The enhanced FAO produced ATP, and it helped cancer cells being resistant to glucose deprivation or hypoxia.^33^ Nevertheless, this process is coupled with the generation of large amounts of reactive oxygen species (ROS), which would arouse chronic oxidative stress. Cancer cells contain a lot of misfolded or injured proteins, resulting in the narrow dynamic range of antioxidant protection system. Hence, under the long-term oxidative stress, cancer cells more easily initiate cell death program than normal cells. Some groups supported that enhanced FAO under normoxic conditions does not affect cancer cell growth; it is detrimental under hypoxia.^17^ In our studies, OA promoted FAO and inhibited cancer cell growth. Hence, roles of FAO for cancer proliferation may be context-dependent or cancer cell-type-specific, and more extensive investigations are warranted to understand the effect of cancer metabolic reprogramming on FAO.

In cancer cells, the balance between lipid synthesis and oxidation is important, which provides both the lipids and the energy required to support the cell growth and survival. Therefore, targeted single link of fatty acid metabolism, and even a single aspect of reprogrammed metabolism of cancer cells, will cause metabolic compensation, and it is not suitable for cancer strategy. Natural product OA regulated the multilinks of fatty acid metabolism, including lipid uptake and transport, as well as fatty acid synthesis and oxidation. Moreover, OA inhibited glycolysis and enhanced oxidative phosphorylation of cancer cells as well.^34^ All these metabolic dysfunctions restricted energy and increased ROS level, which cutoff ‘roads for retreat’ completely and resulted in survival crisis, and even death of cancer cells. OA holds immense potential to be promoted as a novel anticancer candidate through metabolism modulation. Interestingly, in our studies, we not only proved the anti-colon cancer effects of OA but also demonstrated its strong prevention effect for inflammation-associated colorectal cancer. Low dosage intake of OA in daily diet might be a new strategy to reduce the occurrence of colon cancer. Although lovastatin also showed the prevention effects for colon cancer to some degree, its anticancer effects were unsatisfactory. Some side effects in liver will limit the application of statin drugs against cancer. Instead, many reports suggested the low toxicity and selectivity of OA.^35, 36^ Our study proposed an underlying metabolism-modulating mechanism that implicated the antitumor effect of OA and recommended OA as a prospect for improving the current chemotherapeutic strategy for the therapeutic as well as preventative treatment of colon cancer.

## Materials and methods

### Cell culture

The human breast cancer cell line HCT116 was purchased from the Cell Bank of Shanghai Institute of Biochemistry and Cell Biology, Chinese Academy of Sciences (Shanghai, China). HCT116 cells were cultured in McCoy’s 5A medium (Sigma, St Louis, MN, USA) supplemented with 10% heat-inactivated FBS (Sijiqing, Hangzhou, China), 100 U/ml penicillin G and 100 *μ*g/ml streptomycin at 37 °C, 95% relative humidity and 5% CO_2_ with 20% oxygen (normoxia), or 1% oxygen (hypoxia). Conditional culture medium was prepared as below: McCoy’s 5A medium (Sigma, St Louis, MN, USA) supplemented with 10% delipidated FBS and pen–strep as above.

### Lipid (Oil Red O) staining

Remove the medium from the cells and gently wash with PBS. Add Formalin (10%) to each well and incubate for 30 min. Remove formalin and gently wash cells with dH_2_O. Add isopropanol (60%) to each well and incubate for 5 min. Remove isopropanol and add Oil Red O Working Solution to completely and evenly cover the cells. Rotate the plate or dish and incubate for 10–20 min. Remove Oil Red O solution and wash with dH_2_O while viewing under the microscope. LDs appear red.

### Fluorescent measurement of neutral lipids

The intracellular neutral lipid distribution in HCT116 cells was examined by staining the cells with nile red fluorescent dye (Sigma-Aldrich, St. Louis, MO, USA) diluted in Hanks and 20 mM Hepes buffer (HHBS), pH 7. The details are described in the Supplemental Materials. The images were captured with an Olympus FV1000 confocal microscope (Olympus Corporation, Japan) with an excitation wavelength of 486 nm; the emission was measured at 570 nm, following the method of Elsey *et al.*^37^For flow cytometer analysis, cells stained with nile red alone were washed twice with 1 ml of cold FACS buffer and centrifuged at 1 500 r.p.m. at 4 °C for 5 min. All pellets were resuspended with 1 ml PBS + 1% paraformaldehyde and incubated on ice in the dark for 15 min. Cells were washed twice, as described above, and finally resuspended with 1 ml of FACS buffer and detected by a BD FACS (FACSCalibur; Becton Dickinson, Franklin Lakes, NJ, USA).

### Free fatty acid quantitation

Fatty acid concentration was determined in the cell culture medium with a FFA quantification kit (BioVision Research Product, Malpitas, CA, USA) upon extraction.

Fatty acids were extracted by adding 200 ml of dichloromethane to 850 ml of medium. The mixture was vortexed and spinned at 10 000 g for 5 min. The organic phase was collected and vacuum dried for 1 h to remove dichloromethane. Dried lipids were then dissolved in 50 ml of assay buffer provided with the kit and used for the assay.

### Analysis of cell cycle

Cells were pretreated as described in Supporting Information method, and then they were analyzed using a BD FACS can flow cytometer (FACSCalibur; Becton Dickinson, Franklin Lakes, NJ, USA). The PI fluorescence signal peak *versus* the integral was used to discriminate among the S, G0/G1 and G2/M phases of the cell cycle using ModFIT software.

### Colony formation assay

Cells pretreated by OA were plated in a six-well dish at 10 000 cells per well in 0.8% agar in McCoy’s 5A culture medium over a 1.2% agar layer. Plates were further incubated in an incubator for 28 days until colonies were large enough to be visualized. Colonies were stained with 0.01% crystal violet for 1 h and counted under an inverted microscope.

### Real-time PCR analysis

Total RNA was extracted using the TriPure Isolation Reagent (Roche Diagnostics, Mannheim, Germany) and then amplified by polymerase chain reaction (PCR). The primer sets used in the PCR amplification were as follows:

*β*-actin-sense (5′-TCCTTCCTGGGCATGGAGTC-3′),

*β*-actin-antisense (5′-TTCT GCATCCTGTCGGCAATG-3′)

ADPR-sense (5′-CTGCTCACGAGCTGCATCATC-3′)

ADPR-antisense (5′-TGTGAGATGGCAGAGAACGGT-3′

FABP-sense (5′-GCCAGCATCACCATGGTGGACGCTTTC-3′)

FABP-antisense (5′-ATCACCAGTGGATCCAGGTCATGCCTC-3′)

SREBP-sense (5′-CTGGTCTACCATAAGCTGCAC-3′)

SREBP-antisense (5′-GACTGGTCTTCACTCTCAATG-3′)

FASN-sense (5′-CCGAGGAACTCCCCTCAT-3′)

FASN-antisense (3′-GCCAGCGTCTTCCACACT-5′)

CPT1-sense (3′-GTCCCGGCTGTCAAAGACA-5′)

CPT1-antisense 3′-CCGACAGCAAAATCTTGAGCA-5′

The relative gene expressions were analyzed using quantitative RT-PCR with *β*-actin as an internal control.

### Immunofluorescence

Treated HCT116 cells were collected and seeded onto glass coverslips processed for immunofluorescence. The glass coverslips were washed twice with cold PBS for 5 min, fixed with 4%paraformaldehyde for 20 min and incubated with 0.2%Triton X-100 for 5 min at 4 °C. After incubation, the cells were blocked with PBS containing 3% BSA for 1 h, and incubated with anti-HIF1*α* antibody or anti-*β-*cantenin(1 : 200, Cell Signaling Technology, Beverly, MA, USA) overnight. After being washed with PBS containing 0.01% Tween 80, the cells were stained with diamidinophenylindole (DAPI) for 30 min. The images were captured with a confocal microscope (Olympus FV1000, Tokyo, Japan).

### Docking study

Molecular docking study was performed using the Discovery Studio 3.0/CDOCKER protocol. HIF1*α* structure was deposited in protein databank, PDB ID: 2PRG. OA or YC-1 structure was treated with ligand preparation and minimization models in Discovery Studio 3.0 to investigate the spatial binding pattern of OA or YC-1 with HIF1*α*.

### Western blotting

Proteins were isolated using lysis buffer, incubated in SDS buffer, separated on SDS-polyacrylamide gels and electroblotted onto PVDF membranes. Immunoreactive protein bands were detected using an Odyssey Scanning System (LI-COR Inc., Superior St., Lincoln, NE, USA). The following antibodies were used for western blotting: *β*-cantenin, Cyclin B1 and *β*-actin (Santa Cruz Biotechnology) at 1 : 400 dilution; HIF1*α*, FASN, SREBP1, p21, CDK1, p-CDK1 and Wnt signaling pathway proteins (Cell Signaling Technology, Beverly, MA, USA) at 1:800 dilution; and CPT1, FABP3/7 and ADRP (ABclonal Biotech Co., Wuhan, China) at 1:1000 dilution.

### Extraction of cytoplasmic and nuclear fractions

Cells were treated with OA for 36 h. Nuclear and cytosolic protein extracts were prepared using a Nuclear/Cytosol Fractionation Kit (BioVision, Mountain View, CA, USA) according to the manufacturer’s protocol.

### Human colon carcinoma xenograft model

Male athymic BALB/c nude mice(35–40 days old) with body weight ranging from 18 to 22 g were supplied by the Academy of Military Medical Sciences of the Chinese People’s Liberation Army (Certificate No. SCXK-(Army) 2007-004). The animals were kept at 22±2 °C and 55−65% humidity in stainless-steel cages under controlled lights (12 h light per day) and were fed with standard laboratory food and water. Animal care was provided in accordance with the recommendations of the Guide for the Care and Use of Laboratory Animals published by the National Institute of Health, USA.

This experiment was conducted in accordance with the guidelines issued by the State Food and Drug Administration (SFDA of China). Forty nude mice were inoculated subcutaneously with 1 × 10^7^ HCT116 cells into the right axilla. After 12 days of growth, tumor sizes were determined using micrometer calipers. Mice with similar tumor volumes (mice with tumors that were too large or too small were eliminated) were randomly divided into the following five groups (six mice per group): saline control, OA (150 mg/kg, p.o., every 2 days), OA (300 mg/kg, p.o., every 2 days), 5-Fu (40 mg/kg, intraperitoneal (i.p.), every 2 days), and lovastatin (25 mg/kg, p.o., every 2 days). Tumor sizes were measured every 3 days using micrometer calipers, and tumor volume was calculated using the following formula: TV (mm^3^)=*d*^2^ × *D*/2, where *d* and *D* were the shortest and the longest diameters, respectively. Mice were killed on day 21, and tumor tissues were used for immunohistochemistry assay.

### AOM/DSS-induced primary colon cancer model in mice

C57BL/6 male mice, 6–8 weeks old, weighing 18–22 g, were supplied by Shanghai Laboratory Animal Center, China Academy of Sciences (Certificate No. 122). The mice were raised in air-conditioned rooms under controlled lighting (12 h light per day) and provided with food and water at discretion. Animal care and surgery protocols were approved by the Animal Care Committee of China Pharmaceutical University. All animals were treated and used in a scientifically valid and ethical manner.

The AOM/DSS-induced primary colon cancer model was built following the method described in the Professor Ciorba’s studies.^38^ In the beginning of the experiment, the model mice were randomly divided into two main groups and fed by normal diet and high-fat diet. Then each main group was administrated with 200 mg/kg OA (p.o., every 2 days) or 25 mg/kg lovastatin (p.o., every 2 days), respectively. Mice were killed on day 105, and colon tumor tissues were used for immunohistochemistry assay.
