# Supplementary material for: Oroxylin A suppresses the development and growth of colorectal cancer through reprogram of HIF1α-modulated fatty acid metabolism
Source: Cell Death Dis. 2017 Jun 8;8(6):e2865–. doi: 10.1038/cddis.2017.261 (PMC5520917; doi:10.1038/cddis.2017.261)
Supplement: Supplementary Figure Legends [file cddis2017261x1.doc]

**Supplementary Figure Legends**

**Figure S1. The differences of lipid metabolism in colon cancer HCT116 cells cultured under normoxia and hypoxia.** HCT116 cells were cultured in the McCoy’s 5A culture media with 10% FBS and 50 μM sodium palmitate (PA) under normoxia or hypoxia. (A) Cells were stained with oil red or nile red and observed. (B) The intracellular free fatty acid was assayed with the Free Fatty Acid Quantification Kit. (C) Western blot assays were performed for HIF1α, ADRP, FABP7, SREBP1, FASN and CPT1. (D) Quantitative RT-PCR assays were performed for HIF1α and fatty acid metabolism–related proteins, including ADRP, FABP, SREBP1, FASN and CPT1, respectively (E) The colony-formation ability was investigated . Bars, *p<0.05 or **p<0.01.

**Figure S2. The influences of HIF1α on fatty acid metabolism of colon cancer.** HCT116 cells transfected with HIF1α siRNA were cultured in the McCoy’s 5A culture media with 10% FBS and 50 μM PA. (A) Intracellular free fatty acid level was measured. (B) Western blot was performed for HIF1α and fatty acid metabolism–related proteins, including ADRP, REBP1, FASN and CPT1. Bars, *p<0.05 or **p<0.01.

**Figure S3. The influences of FASN on the cell growth of colon cancer.** (A-C) HCT116 cells transfected with FASN siRNA were cultured in the McCoy’s 5A culture media with 10% FBS and 50 μM PA under hypoxic conditions. The cell growth (A) , intracellular free fatty acid level (B) and β-catenin level in cytoplasm and in nucleus (C) were assayed, respectively. (D,E) HCT116 cells transfected with FASN cDNA were cultured in the McCoy’s 5A culture media with 10% FBS and 50 μM PA, and treated with OA for 36 h under hypoxic conditions. The cell growth (D) and the subcellular localization of β-catenin were assayed. Bars, *p<0.05 or **p<0.01.
