# Supplementary figures and images for: Oroxylin A suppresses the development and growth of colorectal cancer through reprogram of HIF1α-modulated fatty acid metabolism
Source: Cell Death Dis. 2017 Jun 8;8(6):e2865–. doi: 10.1038/cddis.2017.261 (PMC5520917; doi:10.1038/cddis.2017.261)

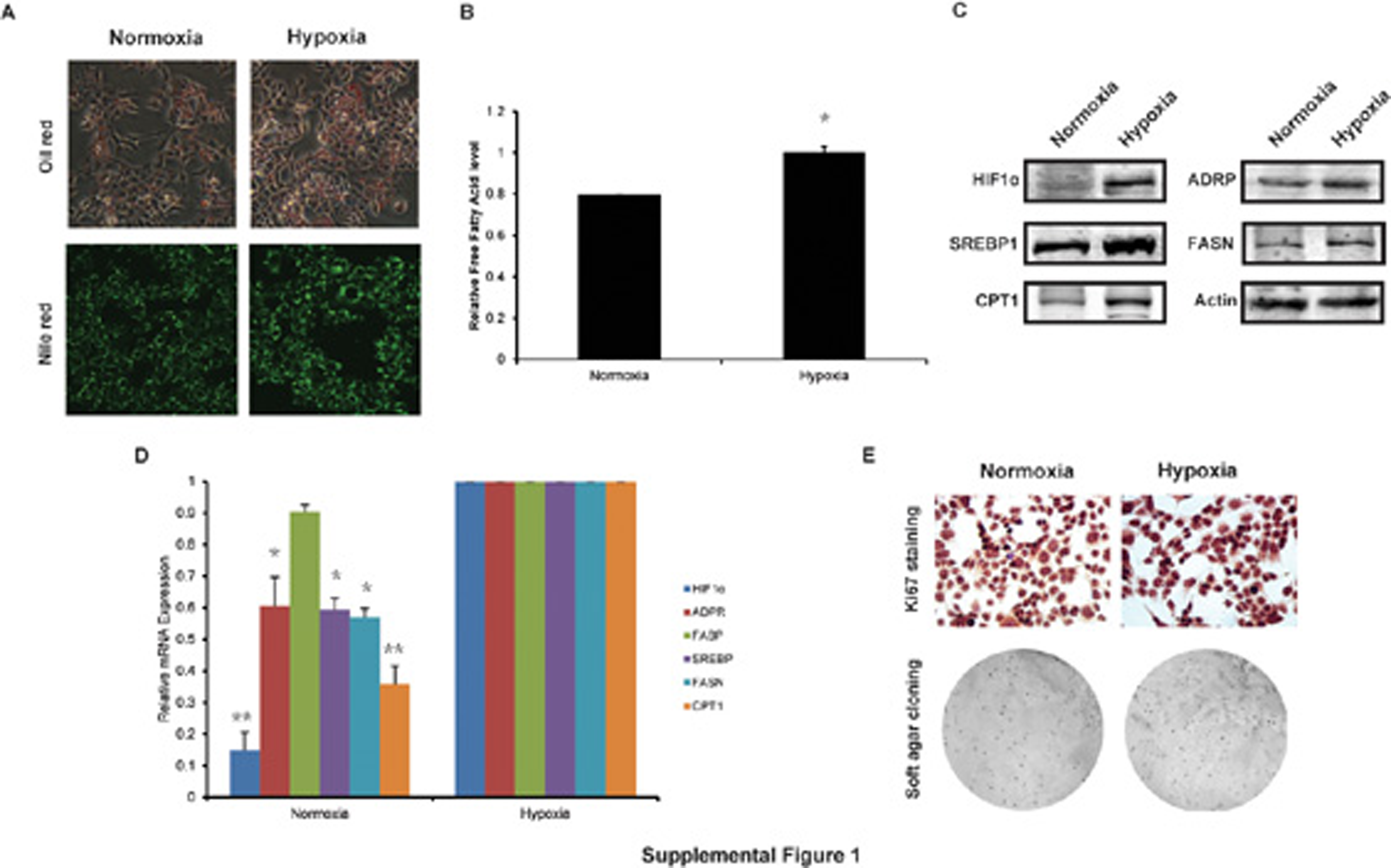

Supplement: Supplementary Figure 1 [file cddis2017261x2.tif]

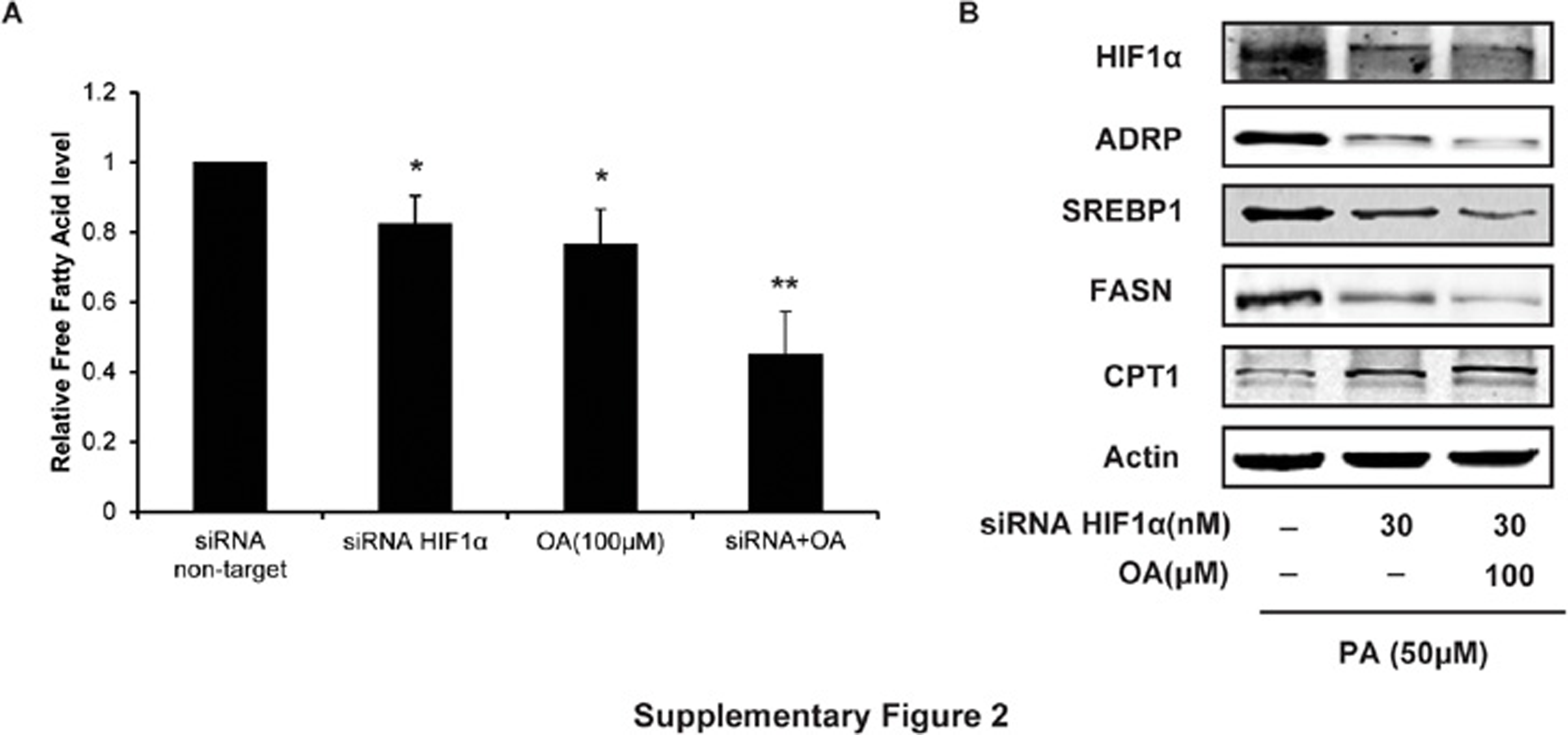

Supplement: Supplementary Figure 2 [file cddis2017261x3.tif]

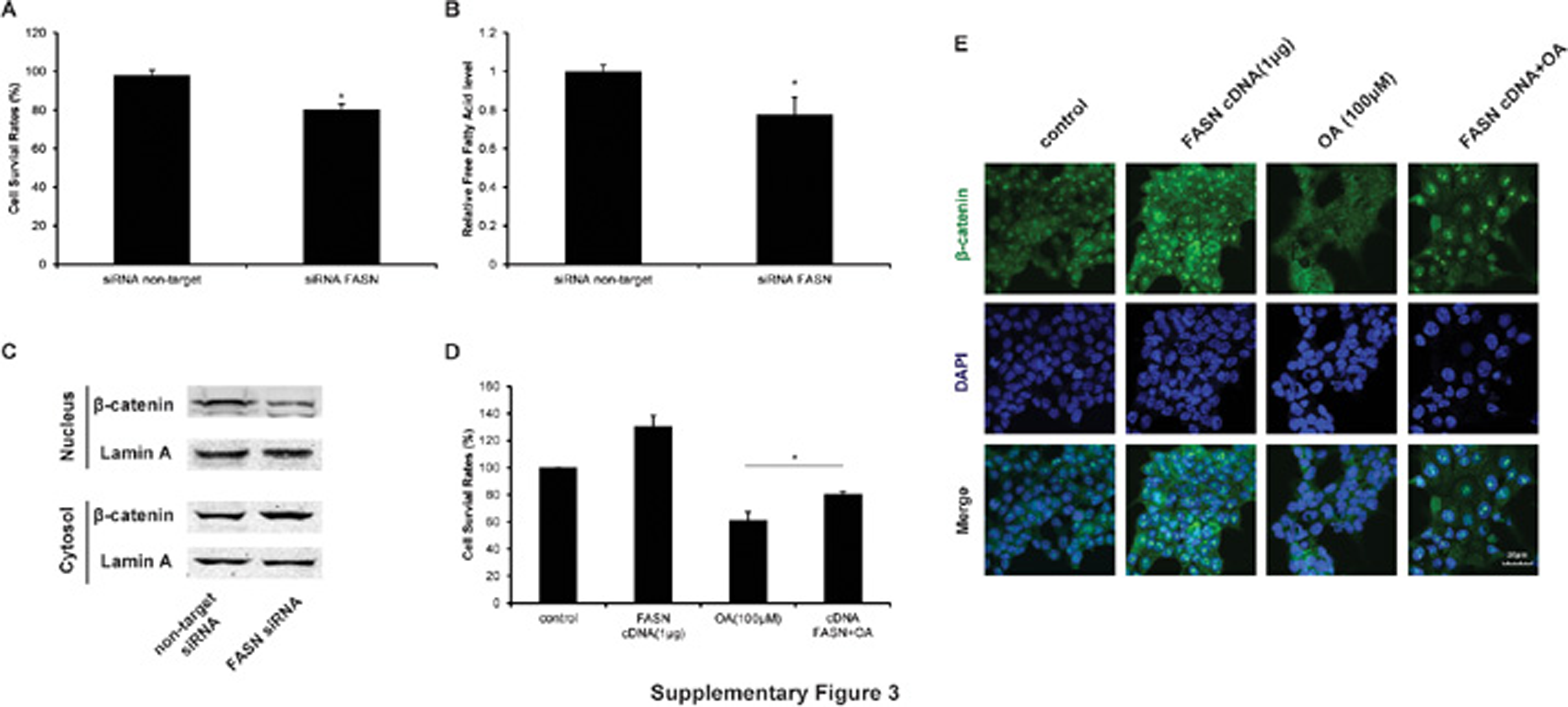

Supplement: Supplementary Figure 3 [file cddis2017261x4.tif]
